# Retaining information from multidimensional correlation MRI using a spectral regions of interest generator

**DOI:** 10.1038/s41598-020-60092-5

**Published:** 2020-02-24

**Authors:** Kristofor Pas, Michal E. Komlosh, Daniel P. Perl, Peter J. Basser, Dan Benjamini

**Affiliations:** 1 National Institute of Biomedical Imaging and Bioengineering, National Institutes of Health, Bethesda, MD 20817 USA; 2 The Department of Biomedical Engineering, University of Texas at Arlington, Arlington, TX 76010 USA; 3 The Eunice Kennedy Shriver National Institute of Child Health and Human Development, National Institutes of Health, Bethesda, MD 20817 USA; 4 The Center for Neuroscience and Regenerative Medicine, Uniformed Service University of the Health Sciences, Bethesda, MD 20814 USA

**Keywords:** Magnetic resonance imaging, Biomedical engineering, Imaging techniques

## Abstract

Multidimensional correlation magnetic resonance imaging (MRI) is an emerging imaging modality that is capable of disentangling highly heterogeneous and opaque systems according to chemical and physical interactions of water within them. Using this approach, the conventional three dimensional MR scalar images are replaced with spatially resolved multidimensional spectra. The ensuing abundance in microstructural and chemical information is a blessing that incorporates a real challenge: how does one distill and refine it into images while retaining its significant components? In this paper we introduce a general framework that preserves the spectral information from spatially resolved multidimensional data. Equal weight is given to significant spectral components at the single voxel level, resulting in a summarized image spectrum. This spectrum is then used to define spectral regions of interest that are utilized to reconstruct images of sub-voxel components. Using numerical simulations we first show that, contrary to the conventional approach, the proposed framework preserves spectral resolution, and in turn, sensitivity and specificity of the reconstructed images. The retained spectral resolution allows, for the first time, to observe an array of distinct ${T}_{1}$−${T}_{2}$−$\langle D\rangle$ components images of the human brain. The robustly generated images of sub-voxel components overcome the limited spatial resolution of MRI, thus advancing multidimensional correlation MRI to fulfilling its full potential.

## Introduction

Biological tissues, particularly within the brain, are highly intricate systems composed of a variety of different materials and structures spanning multiple length scales. Characterization of these different tissues begins on a microscale, each tissue containing an ecosystem of microenvironments. An assortment of cell types, extracellular components, and membranes comprise these microenvironments, all of which perform and participate in a range of chemical processes, yielding unique functionality within the domain. Magnetic resonance imaging (MRI) is a widely applied medical imaging modality for numerous clinical applications. MRI can provide reproducible, noninvasive, and quantitative measurements of tissue, including structural, anatomical and functional information.

Since its introduction in the early 1970s, MRI has been primarily conceived of and used as a radiological scanning tool. Most clinical MRI applications provide images that contain scalar quantities that are averaged across a voxel, which is typically on the order of millimeters. One particular approach to overcome MRI’s limited spatial resolution is to harness this technology’s original designation as a spectroscopic modality. Achieving that goal, a series of advances within the field of porous media nuclear magnetic resonance (NMR)^1–5^ have led to the conception of multidimensional correlation MR^6–13^. This multidimensional approach is a phenomenological representation that makes no assumptions about tissue structure or composition, and jointly encodes and processes different dynamic processes, such as longitudinal (${T}_{1}$) or transverse (${T}_{2}$) relaxation, and diffusion ($D$). The result is a joint multidimensional distribution of these dynamic properties and their correlations, allowing for characterization of heterogeneous systems with increased specificity.

These techniques assume that the acquired signal is made up of a sum of exponenitals, each with their respective ${T}_{1}$, ${T}_{2}$, and $D$. In neuronal tissue, while first order relaxation processes indeed decay in a multiexponential manner, it is well-known that diffusion does not truly behave as a sum of Gaussian processes^14^. As a result, interpretation and modeling of diffusion in neural tissue is an ongoing area of research^15–20^. Nevertheless, modeling of the diffusion signal as a distribution of apparent diffusivities in neuronal tissue can be a way to separate water components based on the range of their apparent mobility^4,21–26^.

Integration of this multidimensional approach within imaging applications was not feasible until recently due to burdensome data requirements causing impractical scan times. The development of the marginal distributions constrained optimization (MADCO) framework^27^ had minimized data requirements, leading to the feasibility of multidimensional correlation imaging. Although the spatial resolution of MRI is low compared with biological tissue local heterogeneity, a spectroscopic approach such as multidimensional MRI provides a multicomponent signature in each voxel, which can be used to report on physical microstructure and chemical composition of a range of tissue types, from animal spinal cord^24,25^ to human placenta^28^.

Use of multidimensional techniques provides a powerful tool for MR analysis; however, it also presents a set of challenges. Through the inclusion of multiple dimensions, there is an increased need to generate a more compact representation of spectral information. The most commonly used method for dimensionality reduction in this context is to compute the relative signal fractions of the spectral components in each voxel and display them as images^29,30^. This dimensionality reduction is achieved by finding spectral regions of interest (sROIs) that contain peaks, and summing (i.e., numerically integrating) over them.

The greatest challenge with generating images from multidimensional spectra is adequately defining the sROIs because in many instances the spectral peaks overlap or somewhat indistinguishable^31^. Moreover, with an imaging volume that easily contains thousands of voxels, a completely automated and robust method to correctly identify the sROI is of paramount importance. Multidimensional MRI is a new and emerging field, and to date the conventional approach to identify sROIs in a large dataset involved compressing the information into a single spectrum by averaging the multidimensional spectra over the entire image^24,28^. Once done, sROIs can be identified manually by the user, or automatically.

We will show in this paper that the conventional approach leads to an unequivocal loss of spectral resolution, and in turn, loss of sensitivity and specificity. We will further propose an automatic sROI generator and demonstrate its ability to preserve the inter-voxel variability and heterogeneity of the multidimensional correlation MRI spectra both on synthetic data, and for the first time, on *ex vivo* human brain.

## Theory

### Multidimensional correlation MR

To obtain a multidimensional distribution of a set of $d$ MR contrasts, $f({x}_{1},\ldots ,{x}_{d})$, the following Fredholm integral of the first kind should be solved,

1 $$m({\beta }_{1},\ldots ,{\beta }_{d})=\int \cdots \int f({x}_{1},\ldots ,{x}_{d})\ {k}_{0}({\beta }_{1},{x}_{1},\ldots ,{\beta }_{d},{x}_{d})\ \,{\rm{d}}{x}_{1}\cdots {\rm{d}}\,{x}_{d}+\epsilon ({\beta }_{1},\ldots ,{\beta }_{d}).$$

The signal, $m$, is acquired with $d$ MR experimental variables, or dimensions, and consequently the density distribution function $f$ is multidimensional. The kernel, or dictionary, which relates the MR parameters to the acquisition variables is noted as ${k}_{0}(\beta ,x)$, and is assumed to be exponential^2,6^, and $\varepsilon (\beta )$ is the experimental noise. We focus here on three MR dimensions, such that $d=3$, and ${x}_{1}={T}_{1}$, ${x}_{2}={T}_{2}$, and ${x}_{3}=\langle D\rangle$, which are encoded by varying the inversion time, ${\beta }_{1}={\tau }_{1}$, the echo time, ${\beta }_{2}={\tau }_{2}$, and the diffusion weighting (DW) parameter, ${\beta }_{3}=b$, respectively. It should be noted that to avoid computational instability and infeasible acquisition time, the diffusion was not characterized using a tensor distribution^32^. Instead, we investigated the orientationally averaged diffusivity, $\langle D\rangle$, encoded by the isotropic generalized diffusion tensor MRI (IGDTI) acquisition protocol^33^. This type of diffusion encoding increases the contrast given by local anisotropy, and is not intended to measure the isotropic diffusion in the system.

Equation 1 can be written with the suitable kernel and parameters as

2 $$m({\tau }_{1},{\tau }_{2},b)=\int \int \int f({T}_{1},{T}_{2},\langle D\rangle )\exp [\,-\,{\tau }_{1}/{T}_{1}-{\tau }_{2}/{T}_{2}-b\langle D\rangle ]\,{\rm{d}}{T}_{1}\,{\rm{d}}{T}_{2}\,{\rm{d}}\,\langle D\rangle +\epsilon ({\tau }_{1},{\tau }_{2},b),$$

while the longitudinal relaxation kernel is in fact a modified version of the conventional kernel, obtained by subtracting the fully recovered data from the data set, and is done to eliminate any signal offset. To solve Eq. 2, the signal is decomposed into a summation of exponential components each with unique MR parameters and amplitude. Because the functional form that relates the MR parameters to the acquisition variables is smooth and continuous, solving a Fredholm integral is an ill-posed problem^34–36^, which means that the solution does not vary smoothly with the data. The strategy we used here to solve Eq. 2 was to transform it to a regularized constrained optimization problem, using ${\ell }_{2}$ regularization to stabilize the solution^1,2^, and applying the marginal distributions constrained optimization (MADCO) framework^27,37,38^ to reduce data requirements. For more details, please refer to the Supplementary Information.

### Automated spectral regions of interest generator

Here we propose a post-processing framework aimed at preserving the spectral information from a spatially resolved multidimensional data. Although conceived for MRI applications, this program could be revised and used for applications in a myriad of fields with high-dimensional spectroscopic data.

The sROI generator is designed to preserve inter-voxel variability and prevent the spectral blurring that is caused (as will be seen here) by using the conventional approach of direct spatial averaging. Our guiding notion is that every spectral component is valuable, regardless of its prevalence or its amplitude in the image and spectral domains, respectively. The proposed method gives equal weight to all spectral components across the image, thus preserving the information they represent.

**Figure 1:**
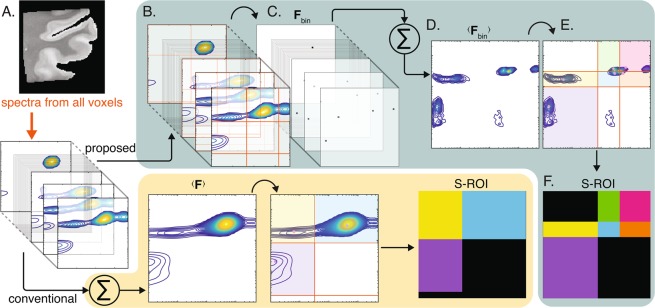
Overview of the proposed method, compared with the conventional approach. (**A**) Each voxel in a given image contains a 2D spectrum. Up to date, the only strategy to process the voxelwise spectra into images was to average them^24,28^, manually identify spectral components, and generate sROIs accordingly (bottom panel). To circumvent the obvious limitations of the standard approach we propose here to first (**B**) identify all possible spectral peaks in each voxel, and then (**C**) apply a threshold and obtain binary voxelwise spectra, ${{\bf{F}}}_{{\rm{bin}}}$. (**D**) The binary spectra are then averaged to yield $\langle {{\bf{F}}}_{{\rm{bin}}}\rangle$, (**E**) which is then used to generate the (**F**) sROIs. The binarization step ensures that even peaks with low prevalence in the image domain are represented in $\langle {{\bf{F}}}_{{\rm{bin}}}\rangle$.

Without loss of generality, we choose to focus on 2D correlation distributions in this work. Figure 1 presents an overview of the proposed approach, compared with the conventional method. Each image voxel contains a 2D correlation distribution, ${\bf{F}}\in {\Re }^{{N}_{{x}_{1}}\times {N}_{{x}_{2}}}$ (Fig. 1A), with projections ${{\bf{f}}}_{{x}_{1}}$ and ${{\bf{f}}}_{{x}_{2}}$.

In essence, we suggest replacing the distribution with a binary map of the significant peaks in each voxel. When averaged across the image, this simple yet effective procedure will result in a distribution that preserves even relatively small spectral components and making them identifiable. To implement this procedure, we chose to use 1D projections because it proved to be robust and easy to implement. However, searching for 2D peaks can be done with any other algorithm, such as feature selection.

Voxelwise, the following steps are taken:

1. Using the projections of ${\bf{F}}$, ${{\bf{f}}}_{{x}_{1}}$ and ${{\bf{f}}}_{{x}_{2}}$ are independently analyzed to locate the 1D spectral peaks.
2. Based on the peaks locations, the intervals in which they reside are then found using the zero crossing of the first derivative of the 1D distributions.
3. Once deduced, these 1D intervals are then used to generate 2D intervals, or "boxes”, which may or may not contain a significant spectral peak (Fig. 1B).
4. The maximal value is found in each of the boxes, and is then compared with a threshold value, ${\varepsilon }_{vox}$. This threshold determines the sensitivity of the peak detection, and is left at the discretion of the user, depending on the application and goals of the particular study.
5. A spectroscopic “center of mass” is calculated in each of the boxes that contained a peak that survived the thresholding step. The center of mass location is replaced with 1, and the rest of the entries with 0.
6. Steps (4) and (5) are repeated for all of the boxes, and result in a binary map of the significant 2D peaks at a given voxel, ${{\bf{F}}}_{{\rm{bin}}}\in {\Re }^{{N}_{{x}_{1}}\times {N}_{{x}_{2}}}$ (Fig. 1C).

Upon completion of these steps in each of the image voxels, the binary peaks maps are averaged across the image domain and normalized to yield $\langle {{\bf{F}}}_{{\rm{bin}}}\rangle$ (Fig. 1D). The last step of this process involves putting $\langle {{\bf{F}}}_{{\rm{bin}}}\rangle$ through steps (1)–(4) (Fig. 1E), which results in the sROIs in the analyzed image (Fig. 1F). Alternatively, $\langle {{\bf{F}}}_{{\rm{bin}}}\rangle$ can be used to manually select sROIs to ensure a more precise outcome. In this work, we used the former approach as a final step (i.e., unsupervised peak finding). The identified sROIs can be used as integral boundaries for 2D integration that would generate signal fraction images of specific spectral components.

The choice of ${\varepsilon }_{vox}$ should be guided by the goal and the context of the study. If small regions in the image domain or alternatively low intensity spectral peaks are of interest, a lower threshold should be set. The lower ${\varepsilon }_{vox}$ is, the more inter-voxel variability is preserved at the cost of false positive detection of peaks. In the current study, ${\varepsilon }_{vox}=0.001$ was chosen based on visual inspection of the images.

### Types of signal-to-noise ratios

There are two types of signal-to-noise ratios (SNR) that should be considered here: (1) acquired signal and (2) spectral SNRs. The first type is usually defined using the acquired image, where the ratio between the average signal intensity within a tissue ROI, and the standard deviation of the signal intensity within a background (i.e., no sample) ROI is taken. In this study,  a typical SNR value was about a 100. Adequate signal SNR is very important to obtain a reliable inversion of Eq. 2. Studies that were dedicated to investigating the effect of SNR on similar inversions concluded that an image SNR of the order of 100 is a sufficient yet realistic value^39,40^.

The second type of SNR is obtained from the spectra (i.e., post-processing), and can be defined as the square of the ratio of the peak and the background amplitudes in the spectrum. In the specific application of multidimensional correlation MR using ${\ell }_{2}$ regularization the resulting spectra are constrained to be smooth, with no wiggles or spikes, which leads to background spectral intensity values on the order of $1{\rm{e}}-9$ (while the peak spectral intensity would be on the order of 0.01). The resulting SNR value is extremely high, approaching infinity, and therefore does not represent a tangible metric to asses or to report.

## Numerical Simulations

When it comes to biological tissue, there is very little "ground truth”. In the context of multidimensional correlation MRI, the ground truth spectral components are unknown and cannot be feasibly determined. If we are to test any proposed method to identify and separate the spectral components, we cannot simply rely on the apparent improvement in the reconstructed spectral images (as will be seen in the next section), but rather test the proposed strategy on a simulated dataset with a known ground truth. In this section we give a non-trivial example of a case in which the conventional approach of spatial averaging fails, while the proposed method works.

**Figure 2:**
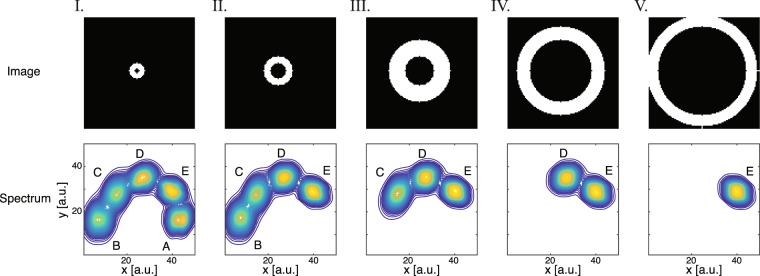
The simulations model. Each column shows a different simulated image-spectrum pair. Pixels in the inner most ring (I) contain all 5 spectral components (**A**–**E**), while pixels in the outer most ring (V) contain only a single spectral component (**E**). The synthetic phantom was created by superimposing all 5 image-spectrum pairs.

We therefore constructed a numerical simulation model, which included five different spectral peaks with distinct means and covariances, detailed in Fig. 2. Each of the peaks was associated with a different spatial coordinate within the synthetic image (i.e., pixel), forming concentric rings in the following manner: pixels in the inner most ring contain all of the spectral components (A–E), while pixels in the outer most ring contain only the spectral component E. As a result, component A is present only in the inner most circle, component B is present only in the the two smallest circles, up to component E, which is present in all of the circles. The 5 image-spectrum pairs in Fig. 2 were then superimposed, resulting in a single multidimensional image that simulated a heterogeneous system with different spectra at different spatial locations. The spectral signal fraction of each component in such an image is weighted by the number of pixels in which it resides, e.g., component A is the least prevalent, and would therefore have the smallest overall signal fraction. Spectral noise was added such that each pixel contained a spectral peak with a slightly different mean and covaraince than the model, normally distributed around the original values with a standard deviation of 1.

**Figure 3:**
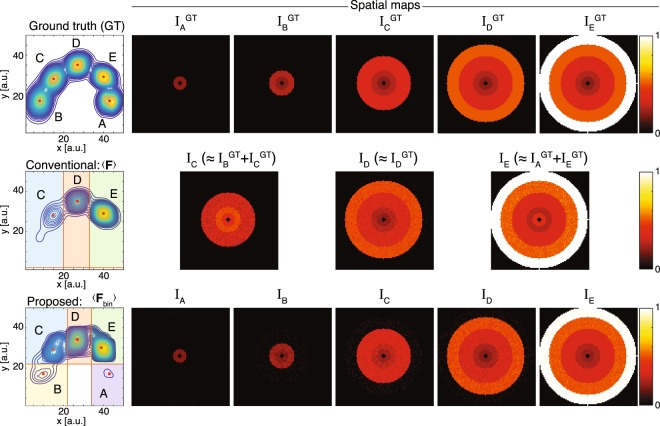
Numerical simulations results. The spectrum on the top row shows the 5 ground truth spectral components unweighted by their prevalence in the image, with their corresponding ground truth spatial images. Results from using the conventional approach to processing spectral imaging data are shown on the center row. $\langle {\bf{F}}\rangle$ is simply an average spectrum across the entire image, and as such, it does not contain components A and B, and therefore cannot be used to correctly reconstruct the ground truth images. The bottom row shows the application of the suggested sROI generator, and its successful identification and reconstruction of the ground truth images.

The results of the numerical simulations are shown in Fig. 3. The ground truth spectrum shows the 5 components, unweighted by their prevalence in the image. To the right of the ground truth spectrum, each image shows a spatial map of an integrated spectral peak, i.e., integration over peaks A–E result in images of their normalized signal fraction, I${}_{\,{\rm{A}}}^{{\rm{GT}}\,}$ to I${}_{\,{\rm{E}}}^{{\rm{GT}}\,}$, respectively. Before testing the conventional and proposed algorithms on the synthetic phantom we should keep in mind that the ability to distinguish between spectral components (i.e., spectral resolution) dictates the specificity of the spatial maps. The challenge in correct and complete reconstruction therefore increases as the prevalence of a given spectral component in the image domain decreases. Thus, for instance, reconstructing I${}_{\,{\rm{A}}}^{{\rm{GT}}\,}$ in our simulations would present the greatest challenge.

The conventional approach of identifying the sROIs was used by averaging the spectra across all image pixels, which resulted in $\langle {\bf{F}}\rangle$ (center row, first column of Fig. 3). Visually, components A and B are not seen; using the automated peak finding algorithm did not improve the outcome. Peaks A and B were not detected because they were simply averaged out, such that $\langle {\bf{F}}\rangle$ no longer contained that spectral information. The process of selecting the sROIs was then used, which resulted in only 3 spectral regions (color coded, C–E) out of the 5 components. Compared with the ground truth, we can see that sROI C contained components C and B, and sROI E contained components E and A. Using these sROIs to integrate over the spectra and generate images resulted in 3 images, and because of the loss of spectral resolution, 2 of them were linear combinations of the correct ground truth images.

We tested our sROI generator on this challenging phantom. Upon visual examination of $\langle {{\bf{F}}}_{{\rm{bin}}}\rangle$, we can see that components A and B were preserved (bottom row, Fig. 3). Using the automated sROI generator, 5 peaks were identified. Integration over those peaks resulted in a robust set of spectral components maps, I${}_{{\rm{A}}}$ to I${}_{{\rm{E}}}$, which was highly similar to the ground truth images, I${}_{\,{\rm{A}}}^{{\rm{GT}}\,}$ to I${}_{\,{\rm{E}}}^{{\rm{GT}}\,}$, with structural similarity indices (SSIM) of $0.80$, $0.82$, $0.75$, $0.84$, and $0.90$, and mean-squared error values of $1.3{\rm{e}}-4$, $3.0{\rm{e}}-4$, $5.8{\rm{e}}-4$, $6.9{\rm{e}}-4$, and $4.8{\rm{e}}-4$, respectively.

## Experimental Results

A human cortical brain specimen was imaged in a 7 T Bruker vertical bore microimaging scanner with an isotropic voxel dimension of 300 $\mu {\rm{m}}$, and a total of 274 acquisitions in the ${\tau }_{1}$−${\tau }_{2}$−$b$ space. The multidimensional correlation MRI resulted in 3 sets of voxelwise 2D spectra, such that each voxel of a 2D image contained ${{\bf{F}}}^{{T}_{1}{\rm{-}}\langle D\rangle }$, ${{\bf{F}}}^{{T}_{2}{\rm{-}}\langle D\rangle }$, and ${{\bf{F}}}^{{T}_{1}{\rm{-}}{T}_{2}}$, leading to 3 sets of 4D information. This multidimensionality is crucial, and as mentioned, it also presents a challenge, especially in extracting significant information and distilling it. We chose to focus on a cortical portion of the human brain because of its known anatomical heterogeneity, and the richness of the microstructural diversity. Similarly to the way the synthetic phantom data was processed, in this section we first processed the correlation spectra using the conventional, spatial averaging approach, and then used the proposed sROI generator to extract images of different spectral components in the brain. For convenience, the MR dimensions were partitioned into time ranges for ${T}_{1}$ and ${T}_{2}$, and mobility ranges for diffusion.

### Conventional approach – spatial averaging

**Figure 4:**
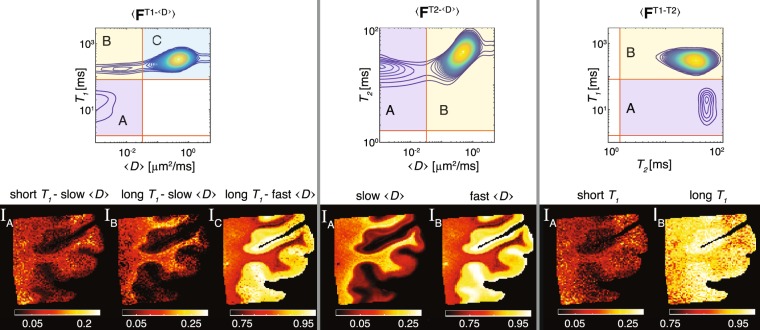
Conventional processing and image reconstruction of multidimensional correlation MRI data. ${T}_{1}$−$\langle D\rangle$, ${T}_{2}$−$\langle D\rangle$, and ${T}_{1}$−${T}_{2}$ distributions averaged across the image were used to locate the sROIs and to generate the signal fraction images (left to right). Note the loss of spectral resolution that resulted in redundancy of the ${T}_{2}$ dimension. Furthermore, many of the reconstructed images appear to be saturated, which implies the blending and smearing of the underlying spectral components.

The top row of Fig. 4 shows spatially averaged ${T}_{1}$−$\langle D\rangle$, ${T}_{2}$−$\langle D\rangle$, and ${T}_{1}$−${T}_{2}$ distributions. In the left column of the Figure, the correlation distribution of diffusivity and longitudinal relaxation averaged across the entire image resulted in 3 spectral components: short ${T}_{1}$−slow $\langle D\rangle$, long ${T}_{1}$−slow $\langle D\rangle$, and long ${T}_{1}$−fast $\langle D\rangle$. These components and their respective sROIs were labeled A–C, which have led respectively to the spatial maps I${}_{{\rm{A}}}$−I${}_{{\rm{C}}}$. Looking at these images, I${}_{{\rm{C}}}$ contained highly saturated regions, which may indicate loss of spectral resolution. In other words, component C in $\langle {{\bf{F}}}^{{T}_{1}{\rm{-}}\langle D\rangle }\rangle$ is likely to be masking several different spectral components.

Looking at $\langle {{\bf{F}}}^{{T}_{2}{\rm{-}}\langle D\rangle }\rangle$, the spectral resolution problem becomes more obvious; contradictory to a large body of research^3,7,41–44^, only 2 distinct diffusion−${T}_{2}$ peaks were captured by the spatially averaged distribution, although at least 3 components are expected from this brain region that contains a mixture of brain gray and white matter (GM and WM, respectively). Furthermore, the A and B sROIs separated the signal contributions exclusively according to the diffusivity dimension, making the ${T}_{2}$ dimension redundant (Fig. 4, center column). Similarly to the case of peak C in $\langle {{\bf{F}}}^{{T}_{1}-\langle D\rangle }\rangle$, here too, component B seems to be smearing over more than one distinct peak.

The dataset that correlated the distribution of the longitudinal and transverse relaxations is perhaps the most extreme demonstration of the importance of our proposed sROI generator (Fig. 4, right column). In this case the ${T}_{2}$ dimension seems to be completely redundant, and the components in $\langle {{\bf{F}}}^{{T}_{1}{\rm{-}}{T}_{2}}\rangle$ are only separable according to their ${T}_{1}$ values. In addition, the resulting long ${T}_{1}$ spatial map, I${}_{{\rm{B}}}$, is obviously saturated and does not provide a very informative contrast.

### New approach – single voxel contributions

**Figure 5:**
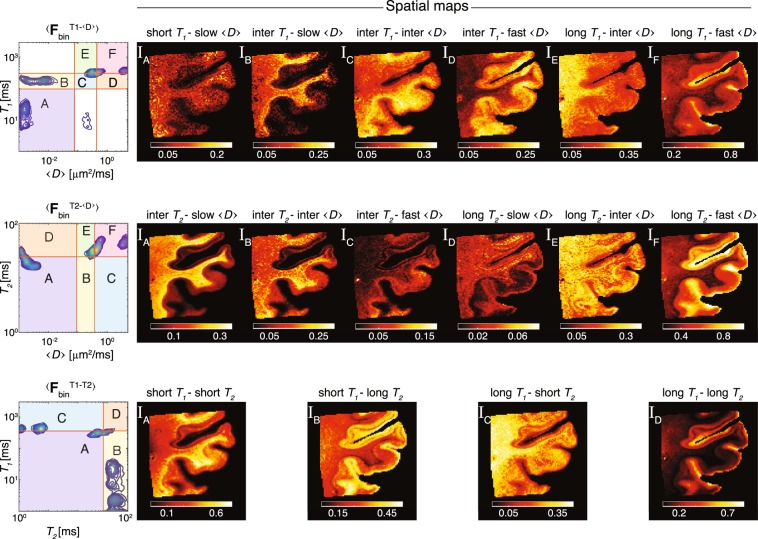
Proposed framework for processing and image reconstruction of multidimensional correlation MRI data. $\langle {{\bf{F}}}_{{\rm{bin}}}^{{T}_{1}{\rm{-}}\langle D\rangle }\rangle$, $\langle {{\bf{F}}}_{{\rm{bin}}}^{{T}_{2}{\rm{-}}\langle D\rangle }\rangle$, and $\langle {{\bf{F}}}_{{\rm{bin}}}^{{T}_{1}{\rm{-}}{T}_{2}}\rangle$ were computed using the proposed algorithm in Fig. 1 (top to bottom). These summarized spectra were then used to identify and define sROIs. Note that compared with the conventional approach, the sROI generator preserved more spectral components, has identified a significantly larger number of multidimensional peaks, and the spatial maps did not contain over-saturated region.

The idea of the proposed automatic sROI generator is to preserve the inter-voxel variability and heterogeneity of the multidimensional correlation MRI spectra. As we saw in the previous section, using a whole image average of the spectra to identify the salient sROIs had led to an unequivocal loss of spectral resolution, and in turn, loss of sensitivity and specificity. In this section, the same 3 sets of voxelwise 2D spectra were processed using the proposed automatic sROI generator algorithm, which yielded the images in Fig. 5. This time, instead of using the spatially averaged spectrum, $\langle {\bf{F}}\rangle$, an average of the binary peaks masks, $\langle {{\bf{F}}}_{{\rm{bin}}}\rangle$, was used according to the proposed algorithm in Fig. 1.

Before examining these results more carefully, we can start by investigating the overall quality and richness of the information in the spatial maps in Fig. 5. The sROI generator preserved more spectral components, has identified a significantly larger number of multidimensional peaks, and the spatial maps did not contain over-saturated regions.

Focusing on the $\langle D\rangle$−${T}_{1}$ correlation (Fig. 5, top row), 6 distinct components were identified (A–F). Looking closely, sROIs A and B are very similar to the ones found using the conventional approach. However, the remaining 4 peaks, which resulted in the spatial maps I${}_{{\rm{C}}}$−I${}_{{\rm{F}}}$, present new information that would have been otherwise remained undetected. These images, which are all complementary to one another (i.e., they sum to 1), reflect specific microenvironments that are distinguished based on apparent water local mobility and longitudinal relaxation, such as long ${T}_{1}$−fast $\langle D\rangle$, etc.

Moving on to the center row of Fig. 5, 6 distinct $\langle D\rangle$−${T}_{2}$ spectral components were identified, leading to 6 spatial maps. Compared with the conventionally obtained spatial images in Fig. 4, in this case the ${T}_{2}$ dimension was not redundant and resulted in a distinct separation to intermediate (inter.) and long ${T}_{2}$ contributions, as expected from previous studies^3,7,12,41–45^. As with $\langle {{\bf{F}}}_{{\rm{bin}}}^{{T}_{1}{\rm{-}}\langle D\rangle }\rangle$, here too the $\langle D\rangle$ dimension, which corresponds to water mobility, was separated into 3 regimes: slow, inter., and fast.

Lastly, we examine the results of the automated sROI generator algorithm on the most challenging case, the ${T}_{1}$−${T}_{2}$ correlation, where the conventional approach was able to resolve only a single dominant spectral component (Fig. 4, bottom row). By taking into account the single voxel contributions that yielded $\langle {{\bf{F}}}_{{\rm{bin}}}^{{T}_{1}{\rm{-}}{T}_{2}}\rangle$, 4 distinct spectral components were observed, leading to the corresponding spatial maps in the bottom row of Fig. 5. The spatial localization of these ${T}_{1}$−${T}_{2}$ components created 4 images with unique contrast and information of the examined brain tissue specimen.

Reproducibility of the sROI generator algorithm was demonstrated by evaluating its consistency across different but consecutive coronal brain slices, in addition to the one shown in Fig. 5. Three adjacent slices were selected, from which the ${T}_{1}$−${T}_{2}$ and the $\langle D\rangle$−${T}_{2}$ datasets were processed. Using the same initialization and parameters for all slices, similar sROIs were identified in all cases, which led to the corresponding ${T}_{1}$−${T}_{2}$ and $\langle D\rangle$−${T}_{2}$ spectral components spatial maps shown in Figs. S1 and S2 in the Supplementary Information. Apart from the anatomical changes that are expected over a length of 1.2 mm, all of the spectral components spatial maps were consistent.

## Discussion

In this study, we developed a novel method to preserve and correctly identify spectral components derived from multidimensional imaging data, thus significantly increasing the effective spectral resolution and specificity of the reconstructed images. Using simulations and experimental data we showed that the proposed framework will outperform the conventional spatial averaging method in most instances, if not all.

The synthetic phantom results demonstrated how the proposed sROI generator is able to preserve spectral resolution, which is also its advantage over the conventional method. Being able to identify spectral components that may be localized in small regions of the image (e.g., pathological tissue), and would have been otherwise averaged out, is extremely important if one wishes to image those abnormal regions.

This development allowed us to apply, for the first time on *ex vivo* human brain, multidimensional correlation MRI. Compared with the conventional approach for processing such data, our results showed the usefulness, robustness and efficiency of the proposed method to automatically generate sROIs. Consistency of spectral images between serial coronal slices of the brain indicates that the the inversion algorithm and the sROI generator framework are stable and reproducible. The significance and impact of this work lies in the interpretation of the multidimensional spectral components images of the brain, shown in Fig. 5. Although it is not the direct subject of this work, a limited interpretation of these images in a biological context may illustrate the potential of the emerging field of multidimensional correlation MRI, and in particular, the use of a robust sROI generator.

Generally speaking, diffusivity is associated with water mobility, and therefore slow $\langle D\rangle$ indicates high microscopic restrictions, or barriers, while faster $\langle D\rangle$ values point to the opposite. In the brain, GM and WM can be distinguished relatively well based on the distribution of $\langle D\rangle$, with GM having spectral components with faster diffusion compared with WM^4,22,46^. The range of ${T}_{2}$ of a specific spectral component can be used to determine whether it is associated with intracellular or extracellular water, the former having longer ${T}_{2}$ than the latter^7,25,43^. Myelin, which is an essential lipid-rich substance that insulates nerve cell axons, can be quantified from the the short ${T}_{1}$^47,48^ and short ${T}_{2}$ components^29,49^.

In our case, each signal fraction image in Fig. 5 contains information from a specific multidimensional spectral component. Based on prior studies, some of these images can be assigned to a physical or biological component with relatively high certainty. The long ${T}_{1}$−fast $\langle D\rangle$ and the long ${T}_{1}$−long ${T}_{2}$ images are likely to be associated with unrestricted bulk water, while the short ${T}_{1}$−short ${T}_{2}$ and the short ${T}_{1}$−slow $\langle D\rangle$ images are presumably associated with myelin. Additionally, clear separation of GM and WM is evident in some of the images, where, for example, the inter ${T}_{2}$−slow $\langle D\rangle$ and long ${T}_{1}$−inter $\langle D\rangle$ images exhibit hyperintensities primarily in WM, while the inter ${T}_{1}$−fast $\langle D\rangle$ image is more specific to the cortical GM. Furthermore, some spectral components images, primarily the short ${T}_{1}$−long ${T}_{2}$ and the long ${T}_{2}$−inter $\langle D\rangle$, capture the heterogeneity of the GM cortical layers, suggesting that multidimensional correlation MRI can be utilized to provide cortical layer-specific information. A complementary neuropathological study of the tissue should be performed to establish a direct correlation between these novel multidimensional MR images and known neuroanatomy. These efforts are currently underway and will be the subject of future publications.

This work proposed and evaluated a robust framework for processing of multidimensional correlation MRI data that preserves spectral resolution while performing the required dimensionality reduction and image reconstruction. We demonstrated that the sROI generator has powerful capabilities for resolving the complex multidimensional spectral signature into separable components using numerical simulations and data from human brain. Furthermore, our work has highlighted the intrinsic flaws of the conventional treatment of multidimensional MRI data. Using a robust processing framework allowed us to apply this new phenomenological imaging modality to generate multiple images of intra-voxel components in the human brain, thus potentially overcoming spatial resolution limitations.

## Materials and Methods

### Tissue preparation

A portion of occipital lobe was obtained from a brain specimen that was derived from the Uniformed Services University/Center for Neuroscience and Regenerative Medicine (USU/CNRM) Brain Tissue Repository collection (U.S. Department of Defense, Bethesda, Maryland, USA). Next of kin provided written consent for participation and brain donation. The tissue archive used have approved procedures for the donation of tissue and storage of clinical information. This study received Institutional Review Board (USU) approval prior to the initiation of the study. All experiments were performed in accordance with the relevant guidelines and regulations. Following formalin fixation, the brain was serially sectioned in the coronal plane. A segment of occipital lobe measuring approximately 5 mm in thickness and containing V1 was then subdissected and used for this study.

### MRI acquisition protocol

The cortical brain specimen was imaged using a 7 T Bruker vertical bore microimaging scanner with a 30 mm quadruple RF coil. A 3D inversion recovery spin-echo diffusion-weighted (DW) echo planar imaging (IR–DWI–EPI) sequence was used with a repetition time of 1200 ms, and the following spatial parameters: field of view (FOV) 33 $\times$ 26 $\times$ 18 ${{\rm{mm}}}^{3}$ and matrix 110 $\times$ 87 $\times$ 60 for isotropic voxel dimension of 300 $\mu {\rm{m}}$. The sample temperature was set at $16.{8}^{\circ }{\rm{C}}$.

For a DW experiment, the spin magnetization decays according to the diffusivity, $D$, due to the experimental parameters, the gradient amplitude, $G$, its duration $\delta$, and separation $\Delta$, which can be summarized in DW parameter, $b={\gamma }^{2}{\delta }^{2}{G}^{2}(\Delta -\delta /3)$, where $\gamma$ is the gyromagnetic ratio. For a ${T}_{1}$−weighted measurement, the spin magnetization returns to thermodynamic equilibrium, followed by an IR experiment with the inversion period, ${\tau }_{1}$, as the governing experimental parameter. Finally, the echo time, ${\tau }_{2}$, governs the decay due to ${T}_{2}$.

The acquisition of multidimensional data was done according to the MADCO framework encoding scheme^25,27^. First, the three 1D distributions of ${T}_{1}$, ${T}_{2}$, and $D$, were estimated, respectively, with the following data acquisition protocols: A 1D ${T}_{1}$-weighted data set ($b=0$, ${\tau }_{2}=11.6$ ms) with 21 logarithmically sampled ${\tau }_{1}$ values ranging from 14.3 to 800 ms by using an IR–DWI–EPI sequence; a 1D ${T}_{2}$−weighted data set ($b=0$) with 20 logarithmically sampled ${\tau }_{2}$ values ranging from 11.6 to 120 ms by using a DWI–EPI sequence. For diffusion encoding, we used the isotropic generalized diffusion tensor MRI (IGDTI) acquisition protocol to achieve an efficient orientationally averaged DW signal^33^ with the following parameters: 21 linearly sampled *b*-values ranging from 400 to 16000 ${{\rm{s}}{\rm{/}}{\rm{mm}}}^{2}$ in 3 directions, 16 linearly sampled *b*-values ranging from 4000 to 16000 ${{\rm{s/mm}}}^{2}$ in 4 directions, and 11 linearly sampled *b*-values ranging from 8000 to 16000 ${{\rm{s/mm}}}^{2}$ in 6 directions, using the efficient gradient sampling schemes in Table 2 of  ^33^. Additional DW parameters were $\delta =4$ ms and $\Delta =15$ ms.

The three 2D distributions of $D$−${T}_{1}$, $D$−${T}_{2}$, and ${T}_{1}$−${T}_{2}$, were estimated, respectively, with the following data acquisition protocols (in conjunction with the *a priori* obtained 1D distributions as constraints): A 2D $D$−${T}_{1}$-weighted data set with 12 sampled combinations of inversion times and *b*-values within the above 1D acquisition range by using an IR–DWI–EPI sequence; a 2D $D$−${T}_{2}$−weighted data set with 16 sampled combinations of echo times and *b*-values within the above 1D acquisition range by using an DWI–EPI sequence; a 2D ${T}_{1}$−${T}_{2}$−weighted data set with 12 sampled combinations of inversion and echo times within the above 1D acquisition range by using an IR-EPI sequence.

## Supplementary information

Supplementary Information.

## Data Availability

The datasets generated and analyzed during the current study are available from the corresponding author on reasonable request.
