# Supplementary material for: Retaining information from multidimensional correlation MRI using a spectral regions of interest generator
Source: Sci Rep. 2020 Feb 24;10:3246. doi: 10.1038/s41598-020-60092-5 (PMC7040019; doi:10.1038/s41598-020-60092-5)
Supplement: Supplementary file 1 — Supplementary Information. [file 41598_2020_60092_MOESM1_ESM.pdf]

Retaining information from multidimensional correlation MRI using a spectral regions of interest generator

Kristofor Pas[1,2], Michal E. Komlosh[3,4], Daniel P. Perl[4], Peter J. Basser[3], and Dan Benjamini[3,4,*]

[1]National Institute of Biomedical Imaging and Bioengineering, National Institutes of Health, Bethesda, MD 20817, USA
[2]The Department of Biomedical Engineering, University of Texas at Arlington, Arlington, TX 76010, USA
[3]The *Eunice Kennedy Shriver* National Institute of Child Health and Human Development, National Institutes of Health, Bethesda, MD 20817, USA
[4]The Center for Neuroscience and Regenerative Medicine, Uniformed Service University of the Health Sciences, Bethesda, MD 20814, USA
[*]dan.benjamini@nih.gov

MR signal processing

Equation 2 can be discretized to vector and matrix notation by writing the continuous distribution $f(T_1, T_2, \langle D \rangle)$ as $\mathbf{f} \in \Re^{\bar{N}_x \times 1}$, with $\bar{N}_x = \prod_{i=1}^{d} N_{x_i}$, and N_{x_i} being the number of spectral components in the ith dimension. The multidimensional signal can be written as $\mathbf{m} \in \Re^{\bar{N}_\beta \times 1}$, with $\bar{N}_\beta = \prod_{i=1}^{d} N_{\beta_i}$, and N_{β_i} being the number of acquired data points in the ith dimension. The kernel can be written as the matrix $\mathbf{K}_0 \in \Re^{\bar{N}_\beta \times \bar{N}_x}$. In this case, Eq. 2 can be written in matrix form as

$$\mathbf{m} = \mathbf{K}_0 \mathbf{f} + \varepsilon, \tag{S1}$$

which can be solved as the following regularized nonnegative minimization problem[1,2]:

$$\hat{\mathbf{f}} = \arg\min_{\mathbf{f} \geq 0} (\|\mathbf{K}_0 \mathbf{f} - \mathbf{m}\|_2^2 + \alpha \|\mathbf{f}\|_2^2). \tag{S2}$$

The regularization term on the right hand side uses an ℓ_2 norm, and is called the Tikhonov regularization[3]. Its use implicitly assumes that the investigated system contains a continuous distribution of physical and chemical microenvironments, which has been standard practice in many previous works[2,4–8]. Importantly, the quadratic nature of the regularization term guarantees the existence of a unique solution to Eq. S2[9]. Selecting the regularization tuning parameter, α, was done using the generalized cross-validation (GCV) method[10].

In addition to the commonly applied spectral constraints, i.e., nonnegativity and limited bandwidth of x, we used here the marginal distributions constrained optimization (MADCO) framework[11,12]. MADCO expands the scope of the applied spectral constraints by using the more accessible marginal distributions to enforce physical constraints on the multidimensional distribution. Given a joint probability density function such as $f(T_1, T_2, \langle D \rangle)$, its 1D projection associated with the ith dimension alone, $f(x_i)$ is defined as

$$f(x_i) = \int \cdots \int f(x_1, \ldots, x_d)\, \mathrm{d}x_1 \cdots \mathrm{d}x_{i-1} \mathrm{d}x_{i+1} \cdots \mathrm{d}x_d, \tag{S3}$$

which, for a discrete system, can be expressed as the following equality

$$\mathbf{f}_{x_i} = \sum_{n \neq i} \cdots \sum_{n \neq i} \bar{\mathbf{F}}_{x_n}, \tag{S4}$$

where $\bar{\mathbf{F}}$ is simply $\mathbf{f}$ reshaped lexicographically into a multidimensional array. The spectral equality constraints in Eq. S4, or its inequality version[12],

$$\|\left(\sum_{n \neq i} \cdots \sum_{n \neq i} \bar{\mathbf{F}}_{x_n}\right) - \mathbf{f}_{x_i}\|_2 \leq \sigma_i, \tag{S5}$$

can be used as a set of additional spectral constraints, thus vastly reducing the required amount of data and significantly improving the inversion's stability[12–15]. Using MADCO requires independently encoding and estimating the lower dimensional distributions, which leads to a "hierarchical" encoding scheme, such as the one used in this study.

For all types of 2D correlations, Eq. S2 subject to the constraints in Eq. S5 was solved voxelwise with $N_{T_1} = N_{T_2} = N_{\langle D \rangle} = 50$, and σ_i was taken as the standard deviation of the noise (as determined after complete signal decay) normalized by the unattenuated signal. All data processing was performed with in-house code written in MATLAB (The Mathworks, Natick, MA). To resolve the 2D spectra subject to the MADCO constraints we used CVX, a package for specifying and solving convex programs[16,17].

Reproducibility and consistency

The reproducibility of the sROI generator framework can be demonstrated by evaluating its consistency across different but consecutive coronal brain slices. Three 300 μm thick slices adjacent to the analyzed image slice in Fig. 5 were selected, from which the T_1-T_2 and the T_2-$\langle D \rangle$ datasets were processed.

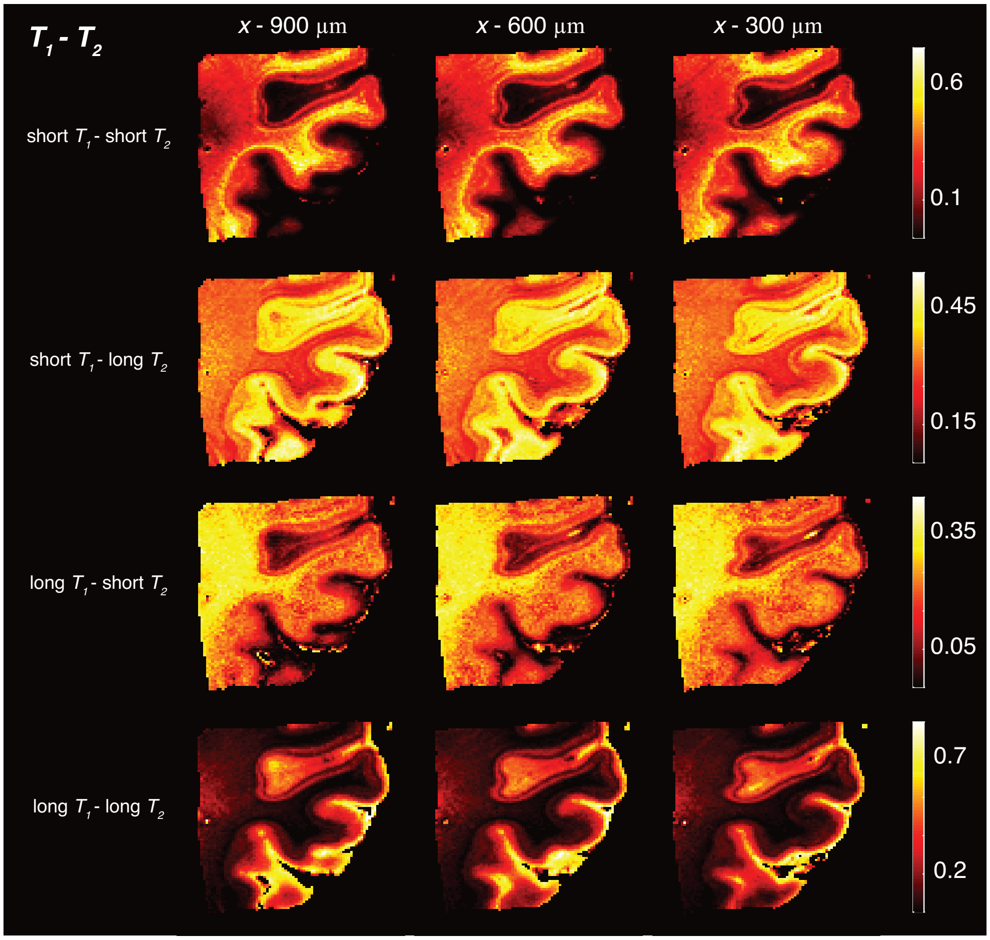

Figure S1. T_1-T_2 spectral components spatial maps derived using the proposed sROI generator framework. Three serial coronal slices adjacent to the original slice location, x, were processed.

Using the same initialization and parameters for all slices, similar sROIs were identified in all cases, which led to the corresponding T_1-T_2 and T_2-$\langle D \rangle$ spectral components spatial maps shown in Figs. S1 and S2. These should be compared with the spatial maps shown in Fig. 5 in the main text. Apart from the anatomical changes that are expected over a length of 1.2 mm, all of the spectral components spatial maps seem fairly consistent. This consistency indicates that the the inversion algorithm and the sROI generator framework are stable and reproducible.

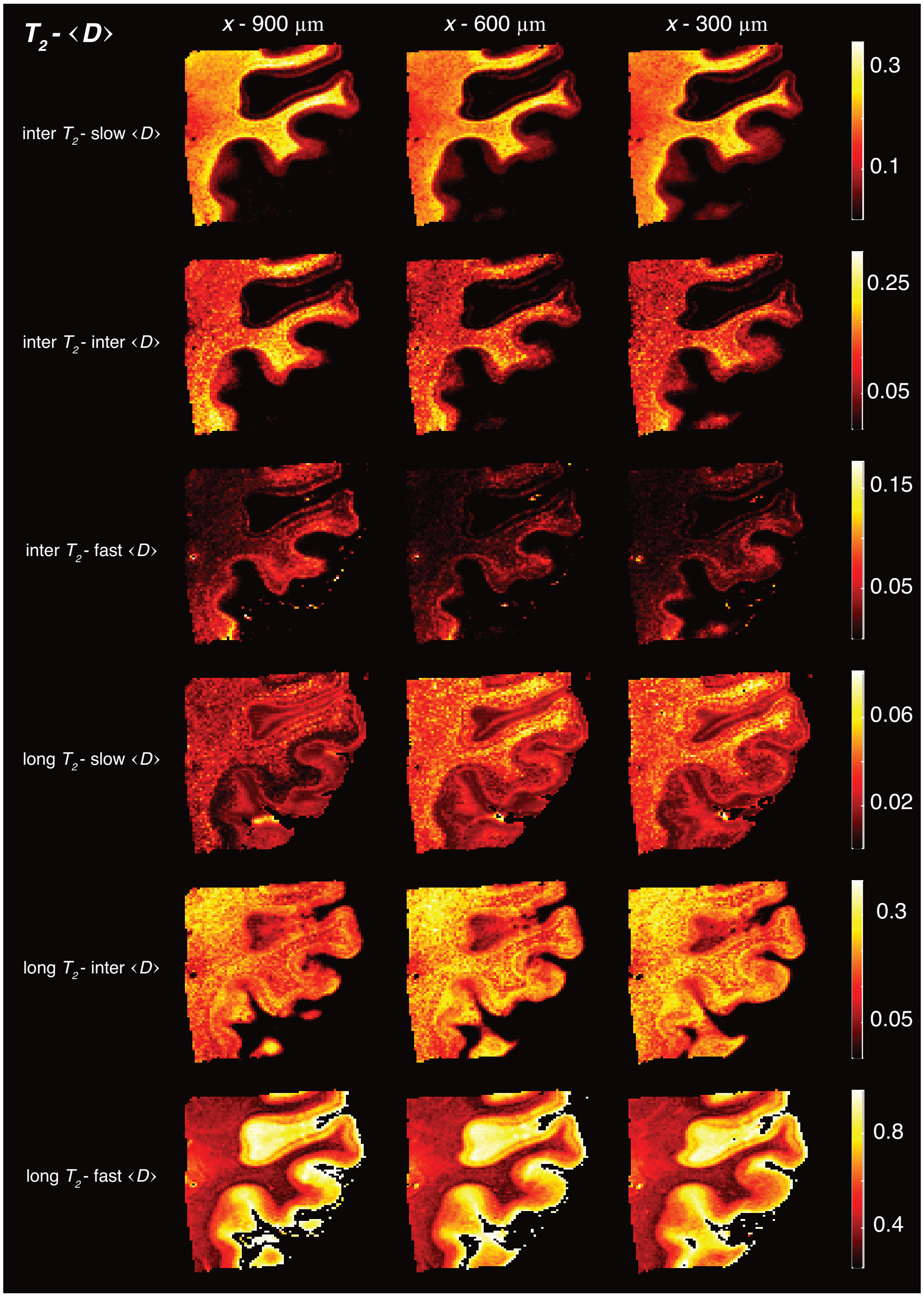

Figure S2. T_2-$\langle D \rangle$ spectral components spatial maps derived using the proposed sROI generator framework. Three serial coronal slices adjacent to the original slice location, x, were processed.

References

1. Provencher, S. W. A constrained regularization method for inverting data represented by linear algebraic or integral equations. *Comput. Phys. Commun.* **27**, 213–227 (1982).
2. Kroeker, R. M. & Mark Henkelman, R. Analysis of biological NMR relaxation data with continuous distributions of relaxation times. *J. Magn. Reson. (1969)* **69**, 218–235 (1986).
3. Tikhonov, A. & Arsenin, V. *Solutions of Ill-Posed Problems* (Winston, New York, 1977).
4. Menon, R. S. & Allen, P. S. Application of continuous relaxation time distributions to the fitting of data from model systmes and excised tissue. *Magn. Reson. Medicine* **20**, 214–227 (1991).
5. Fordham, E., Sezginer, A. & Hall, L. Imaging Multiexponential Relaxation in the (y, LogeT1) Plane, with Application to Clay Filtration in Rock Cores. *J. Magn. Reson. Ser. A* **113**, 139–150 (1995).
6. Pfeuffer, J., Provencher, S. W. & Gruetter, R. Water diffusion in rat brain in vivo as detected at very largeb values is multicompartmental. *Magn. Reson. Mater. Physics, Biol. Medicine* **8**, 98–108 (1999).
7. Ronen, I., Moeller, S., Ugurbil, K. & Kim, D.-S. Analysis of the distribution of diffusion coefficients in cat brain at 9.4 T using the inverse Laplace transformation. *Magn. Reson. Imaging* **24**, 61–68 (2006).
8. Benjamini, D., Komlosh, M. E., Holtzclaw, L. A., Nevo, U. & Basser, P. J. White matter microstructure from nonparametric axon diameter distribution mapping. *NeuroImage* **135**, 333 – 344 (2016).
9. Venkataramanan, L., Song, Y.-Q. & Hürlimann, M. D. Solving Fredholm integrals of the first kind with tensor product structure in 2 and 2.5 dimensions. *IEEE Trans. Signal. Process.* **50**, 1017–1026 (2002).
10. Golub, G. H., Heath, M. & Wahba, G. Generalized Cross-Validation as a Method for Choosing a Good Ridge Parameter. *Technometrics* **21**, 215 (1979).
11. Benjamini, D. & Basser, P. J. Joint radius-length distribution as a measure of anisotropic pore eccentricity: An experimental and analytical framework. *The J. Chem. Phys.* **141**, 214202 (2014).
12. Benjamini, D. & J., B. P. Use of marginal distributions constrained optimization (MADCO) for accelerated 2D MRI relaxometry and diffusometry. *J. Magn. Reson.* **271**, 40–45 (2016).
13. Benjamini, D. & Basser, P. J. Magnetic resonance microdynamic imaging reveals distinct tissue microenvironments. *NeuroImage* **163**, 183–196 (2017).
14. Benjamini, D., Komlosh, M. E. & Basser, P. J. Imaging local diffusive dynamics using diffusion exchange spectroscopy MRI. *Phys. Rev. Lett.* **118**, 158003 (2017).
15. Benjamini, D. & Basser, P. J. Towards clinically feasible relaxation-diffusion correlation MRI using MADCO. *Microporous Mesoporous Mater.* **269**, 93–96 (2018).
16. Grant, M. C. & Boyd, S. P. Graph Implementations for Nonsmooth Convex Programs. In *Recent Advances in Learning and Control*, 95–110 (Springer London, London, 2008).
17. CVX Research. CVX: Matlab Software for Disciplined Convex Programming, version 2.0 (2012).
